# An mRNA-based rabies vaccine induces strong protective immune responses in mice and dogs

**DOI:** 10.1186/s12985-022-01919-7

**Published:** 2022-11-12

**Authors:** Jianglong Li, Qi Liu, Jun Liu, Xiaohong Wu, Yixin Lei, Shuang Li, Danhua Zhao, Zhi Li, Liping Luo, Sophia Peng, Yingrao Ou, Hong Yang, Jing Jin, Yuhua Li, Yucai Peng

**Affiliations:** 1 Liverna Therapeutics Inc., Zhuhai, 519000 Guangdong China; 2 Department of Arboviral Vaccine, National Institutes for Food and Drug Control, Beijing, 100000 China; 3 Rice University, Houston, TX 77001 USA

**Keywords:** Rabies, mRNA vaccine, RABV-G, Viral challenge study, LVRNA001

## Abstract

Rabies is a lethal zoonotic disease that is mainly caused by the rabies virus (RABV). Although effective vaccines have long existed, current vaccines take both time and cost to produce. Messenger RNA (mRNA) technology is an emergent vaccine platform that supports rapid vaccine development on a large scale. Here, an optimized mRNA vaccine construct (LVRNA001) expressing rabies virus glycoprotein (RABV-G) was developed in vitro and then evaluated in vivo for its immunogenicity and protective capacity in mice and dogs. LVRNA001 induced neutralizing antibody production and a strong Th1 cellular immune response in mice. In both mice and dogs, LVRNA001 provided protection against challenge with 50-fold lethal dose 50 (LD_50_) of RABV. With regards to protective efficiency, an extended dosing interval (14 days) induced greater antibody production than 3- or 7-day intervals in mice. Finally, post-exposure immunization against RABV was performed to evaluate the survival rates of dogs receiving two 25 μg doses of LVRNA001 vs. five doses of inactivated vaccine over the course of three months. Survival rate in the LVRNA001 group was 100%, whereas survival rate in the inactivated vaccine control group was only 33.33%. In conclusion, these results demonstrated that LVRNA001 induced strong protective immune responses in mice and dogs, which provides a new and promising prophylactic strategy for rabies.

## Introduction

Rabies is an ancient zoonosis of the central nervous system caused by the rabies virus (RABV) that affects numerous species of warm-blooded animals [1, 2]. Clinically, RABV infections manifest as neuronal dysfunctions that almost inevitably lead to death [3]. Approximately 59,000 people die of rabies annually worldwide, with higher incidences in Asia and Africa [4]. RABV is a non-segmented negative-strand RNA virus of the genus *Lyssavirus*, family *Rhabdoviridae* [5, 6]. The RABV genome is composed of five genes encoding the following proteins: nucleoprotein (N), phosphoprotein (P), matrix protein (M), glycoprotein (G), and RNA-dependent RNA polymerase (RdRp; also termed large protein, L) [7]. Among these viral proteins, G is the only protein that is glycosylated and present in the viral envelope [8]. RABV-G attaches to cellular receptors, such as neural cell adhesion molecules [9] and low-affinity nerve growth factor receptor (p75NTR) [10], and facilitates the entry of virus particles into host cells by fusion with the cellular membrane [11, 12]. The efficient binding of RABV-G to putative host cell receptors ensures virus uptake and promotes virulence [13, 14]. In this way, RABV-G plays an essential role in RABV’s transsynaptic spread throughout the central nervous system [13, 14]. RABV-G is also relevant to the immune response against RABV. As the only protein exposed on the surface of the virion, RABV-G has been reported to be the major target for neutralizing antibodies [15–17] and vaccine development.

There are currently no effective treatments for rabies. However, as RABV can stay at the entry site of infection for days or weeks before arriving at the central nervous system and causing symptoms, immunizations either prior to or soon after exposure can be an effective strategy against the disease. Ideal rabies vaccines that provide successful postexposure prophylaxis should rapidly stimulate potent protective immune responses [18]. Classic inactivated vaccines remain the main rabies vaccines for human on the market, which can provide immune protection when administered pre-exposure or promptly post-exposure, but 4–5 doses are required to achieve protective immunity [18, 19]. Vaccines made from live attenuated viruses could trigger long-lasting immunity with a single dose, but safety concerns, especially the possibility of reverting to pathogenic wild-types or even recombination with other live agents, cannot be ruled out. Oral vaccination of dogs with recombinant rabies virus vaccines was also attempted but with limited understanding of detailed mechanism [20]; There was report that a commercial vaccinia-rabies glycoprotein (V-RG) recombinant virus vaccine failed to provide protection in skunks and dogs when administrated orally as a single dose, and caused severe skin inflammation in humans who occasionally came in contact with the baits [20–22]. Collectively, the development of alternative, cost-effective vaccines that would induce sustained immunity after less dose inoculation and could ideally prevent virus from infecting the CNS is warranted.

mRNA, or messenger RNA, technology is a recent advent in the treatment of infectious diseases and cancer [23, 24]. The mRNA vaccine field has advanced rapidly in the past few years [25], with the Pfizer/BioNTech and Moderna COVID vaccines at their spearhead conferring an efficacy rate of over 90% in clinical stages [26, 27] and other vaccines against viral diseases such as influenza and Ebola underway in many countries [28]. These successes may be due in part to the fact that mRNA vaccines, as a genetic vaccine format, utilize no living virus material and therefore do not run the safety risk of pathogenicity reversion and possible infection [25]. Moreover, mRNA vaccines have induced balanced and enduring immunity in antitumor and prophylactic applications [29–31]. From a manufacturing perspective, mRNA vaccines are also advantageous in that they are easy to develop and purify [32].

In the past few years, non-replicating mRNA-based rabies vaccines with exclusively unmodified nucleosides have been attempted, phase I clinical studies demonstrated that the vaccine candidates induced boostable functional antibodies against RABV-G, and were generally safe with a reasonable tolerability profile [33, 34]. In the current study, we developed a non-replicating mRNA vaccine encoding RABV-G and demonstrated its protective efficacy in mice and dogs. Our results imply that an mRNA vaccine encoding RABV-G can be a prophylaxis for rabies infections.

## Materials and methods

### Cell and viruses

BHK-21 cells were cultured with Dulbecco’s modified Eagle’s medium (DMEM) containing 10% FBS at 37 °C with 5% CO_2_. RABV strain CVS-24 (GenBank: ADR03123.1) was provided by the Institute of Animal Health, Guangdong Academy of Agricultural Sciences (Guangzhou, China). RABV strain BD06 (GenBank: ACB38373.1) was provided by the Institute of Military Veterinary Medicine, Academy of Military Medical Sciences (Changchun, China). BD06 strain could cause 80% mortality in unvaccinated dogs after challenge [35] and is responsible for most rabies cases in humans and dogs in China [36].

### Vaccines

mRNA vaccines were produced based on the Liverna Therapeutics platform (China patent ZL201911042634.2). The mRNA molecules included a 5’ cap structure, a 5’ UTR, an ORF, a 3’ UTR and a poly(A) tail. The ORF in this study encodes the glycoprotein (RABV-G) of the CTN-1 strain (GenBank: ACR39382.1), which has been used for production of human rabies vaccine in China and is Chinese domestic isolates [37, 38]. Three different mRNA constructs were developed: RABV G-A, RABV G-B, and RABV G-C. The RABV G-A sequence consisted of a 5’ UTR, the ORF of RABV-G, a 3’ UTR and a 64A + 36C + histone stem loop. RABV G-B was an optimized RABV G-A sequence with a different ORF. RABV G-C was identical to RABV G-B with the exception of its poly(A) tail. Unless specifically noted, RABV G-C was the sequence of our mRNA vaccine, named LVRNA001.

mRNAs were produced by in vitro transcription (IVT). DNA templates were linearized from plasmids containing the open reading frames flanked by 5’/ 3’UTR and Poly-A sequences. IVT reactions were performed using DNA templates, an optimized T7 RNA polymerase (Novoprotein Scientific Inc., China), NTP and Cap GAG m7G(5′)ppp(5′ (2′-OMeA)pG (Jiangsu Synthgene Biotechnology Co, China). The reaction was terminated by addition of DNase I (Novoprotein Scientific Inc., China). mRNAs were purified using Oligo-dT affinity column (Sepax Technologies, Inc., China) and Tangential Flow Filtration (TFF, Repligen Corporation, America). Microfluidic capillary electrophoresis (Fragment Analyzer systems 5200, Agilent) was used to assess RNA integrity, and the characterization including concentration, pH, residual DNA, proteins, and dsRNA impurities of the solution were also performed. To prepare the mRNA vaccines, purified mRNAs were encapsulated in LNPs according to a modified procedure wherein cholesterol, 1,2-distearoyl-sn-glycero-3-phosphocholine (DSPC), a polyethylene glycol-lipid and a cationic lipid was rapidly mixed with an aqueous solution containing mRNA. Then, the analytical characterization of product was carried out, including the determination of particle size and polydispersity, encapsulation, pH, endotoxin, and bioburden. The final products were lyophilized into powder in 2 ml glass vials. Sterile water (1 ml) was added to each vial to produce a 25 μg/ml (mRNA) solution before use. Appropriate volumes were taken from the vial for animal injection according to the experimental plan.

Inactivated vaccines were purchased from an animal hospital (Rabvac®, produced by Boehringer Ingelheim Vetmedica, Inc. Lot number 4150466A, labelled potency is 1 dose ≥ 2.0 IU) or a domestic vaccine manufacturer (rabies vaccine made from aGV strain for human use, freeze-dried, labelled potency is 1 dose ≥ 2.5 IU).

### Protein expression

Protein expression was measured using flow cytometry analysis as previously described [39]. Briefly, HEK293 cells were transfected with either RABV-G mRNA or Luc mRNA (negative control) for 24 h. Cells were then collected and stained with a monoclonal mouse anti-rabies antibody (HyTest Ltd, Turku, Finland) and an FITC-labeled goat anti-mouse IgG (Life Technologies GmbH, Darmstadt, Germany). Flow cytometric analysis of FITC-positive cells confirmed protein expression.

### Transmission electron microscopy (TEM)

The morphology of the nanoparticles was analyzed using TEM. Briefly, a drop of aqueous nanoparticle sample was dropped onto a carbon-coated copper grid. Subsequently, the grid was air-dried completely at room temperature. TEM micrograph images were captured at an accelerating voltage of 80 kV.

### Mouse and dog immunization and viral challenge

BALB/c mice (~ 6 weeks old, 20–25 g) were purchased from the Institute of Animal Health, Guangdong Academy of Agricultural Sciences (Guangzhou, China). Dogs (~ 3 months old beagles, 5–6 kg) were obtained from the Institute of Military Veterinary Medicine, Academy of Military Medical Sciences (Changchun, China). All experiments were approved by the Research Ethics Committee of the College of Animal Health, Guangdong Academy of Agricultural Sciences, and the Research Ethics Committee of the College of the Institute of Military Veterinary Medicine, Academy of Military Medical Sciences (IACUC of AMMS-11–2021-19). Mouse immunizations were carried out according to the methods described previously [40], LVRNA001 (0.2–5 μg) or 0.1 dose (1 dose ≥ 2.0 IU) of Rabvac® were intramuscularly (i.m.) injected into the thigh of hindlimb once (0d) or twice (0d/7d); 14 days post immunization (dpi), blood samples were collected for antibody tests and mice were injected with viruses (strain CVS-24, 50-fold LD_50_ pre-determined by the Institute of Animal Health, Guangdong Academy of Agricultural Sciences for mouse in this experiment) intracerebrally, followed by observation of animal survival and last round of blood sample collection on day 21 post challenge for neutralization antibody test.

For dog challenge studies, LVRNA001 (5 or 25 μg, 0d/7d or 0d/7d/21d) or an inactivated vaccine made for human use (1 dose, ≥ 2.5 IU, 0d/3d/7d/14d/28d) were i.m. injected into the lateral thigh of hind limb. Blood samples were collected on days 0, 7, 9, 11, 13, 35 post first injection for antibody tests. Viruses (strain BD06) were given to the test dogs on day 35 by i.m. injection to the biceps femoris of the hind limb at 50-fold LD_50_ (pre-determined by the Institute of Military Veterinary Medicine, Academy of Military Medical Sciences for dog in this experiment). Observation of animal survival continued through 3 months after challenge, when blood samples were collected from survived dogs for neutralization antibody test.

### Post-exposure immunization against RABV in dogs

Dogs were intramuscularly injected with 50-fold LD_50_ of virulent RABV-BD06 strain in the biceps femoris of the hind limb. Six hours after challenge infection, dogs were i.m. injected with LVRNA001 or inactivated vaccine. Immunization procedures and experimental steps were the same as pre-exposure protocols shown above.

### Enzyme-linked immunosorbent assays (ELISA)

Spleen lymphocytes from mice in each immunization group were collected and resuspended in RPMI 1640 medium containing 10% fetal bovine serum (FBS). Then, 4 × 10^6^ cells were seeded into a 24-well flat-bottom tissue culture plate and incubated with 5 μg of a synthesized peptide library of RABV-G for 72 h at 37°C. The cell supernatants were collected, the interferon (IFN)-γ and interleukin (IL)-4 levels were measured using ELISA kits (Neo Bioscience, China) according to the manufacturer’s instructions.

### Intracellular cytokine staining

TNF-α-producing CD3^+^/CD4^+^ or CD3^+^/CD8^+^ T cells from mouse spleen lymphocytes (collected 7 days after booster immunization) were analyzed using flow cytometry. Spleen lymphocytes (4 × 10^6^ cells/ml) were seeded into a 24-well flat-bottom tissue culture plate and incubated with 5 μg of a synthesized peptide library of RABV-G for 72 h at 37°C. Cells were then stained with the following antibodies at 4°C for 20 min: CD8-FITC (1:200), CD3-PerCP-Cy5.5 (1:200), CD4-BV605 (1:200) (all from BD Biosciences, Heidelberg, Germany), and TNF-α-PE (1:100) (BioLegend, San Diego, CA, USA). The fluorescence signals from staining were measured by flow cytometry, and data analysis was conducted using FlowJo software (Tree Star, Inc., Ashland, USA).

### RABV-G-specific immunoglobulin measurements

Mouse serum samples were collected and scanned for RABV-G-specific immunoglobulin using a commercially available RABV antibody detection kit (Synbiotics Corp, France) following the manufacturer’s instructions. A positive antibody titer was recognized as OD_450_ > 0.2.

### Serum neutralization assay

Viral neutralizing antibody (VNA) titers against RABV were determined by fluorescent antibody virus neutralization (FAVN) tests according to standards set forth by the World Organization for Animal Health. Serum samples were serially diluted into a 96-well plate along with WHO reference serum diluted to 0.5 IU/ml. A 50 μL suspension containing the 50% tissue culture infectious dose (TCID_50_) of challenge virus standard strain 11 (CVS-11, obtained from Chinese National Institutes for Food and Drug Control) was added to each well, and the plates were incubated at 37°C for 1 h. BHK-21 cells were cultured with DMEM containing 10% FBS and then added to the wells, and the plates were incubated at 37°C in a humidified incubator with 5% CO_2_ for 48 h. Afterward, the cells were washed and fixed in 80% cold acetone for 30 min. Last, the cells were covered with a RABV-specific monoclonal antibody (KPL, Gaithersburg, MD, USA) followed by FITC-conjugated goat anti-mouse IgG (KPL, Gaithersburg, MD, USA). Fluorescent signals in each well were detected with a fluorescence microscope.

### Direct immunofluorescence detection of RABV in mouse brain

Mouse brain tissue was collected and fixed with 4% paraformaldehyde. Tissue samples were blocked with bovine serum albumin (BSA) and incubated with a RABV-specific primary antibody for 1 h. Samples were then washed with an FITC-conjugated secondary antibody and imaged with a fluorescence microscope.

### Statistical analysis

Data are presented as the mean ± standard deviation (SD). Statistical analyses were conducted using GraphPad Prism 6.0 software. Comparisons between groups were performed with one-way ANOVA followed by Tukey’s test. A p value less than 0.05 was considered statistically significant.

## Results

### Design and expression of the mRNA vaccine encoding RABV-G

**Fig. 1:**
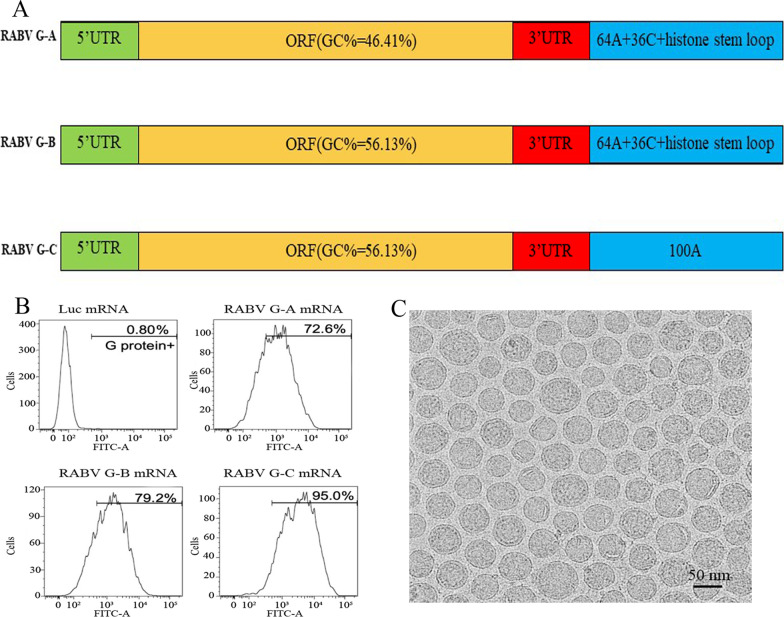
Design and expression of the mRNA vaccine encoding RABV-G. **A** A panel of RABV-G mRNA antigens was designed. **B** HEK293 cells were transfected with RABV-G mRNA or Luciferase mRNA (negative control) for 24 h. Cells were collected and used to measure RABV-G expression by flow cytometry. **C** Spherical nanoparticles were observed under an electron microscope

RABV-G is the major viral antigen that induces neutralizing antibody production in infected species [41, 42]. To both improve its expression and stabilize its desired conformation, we designed a panel of RABV-G mRNA antigens: RABV-G A, RABV-G B, and RABV-G C (Fig. 1A). We then compared the antigen expression induced by these optimized mRNA constructs. Flow cytometry analysis revealed that the expression of RABV-G induced by RABV-G C (95.0%) was the highest among the three mRNA constructs (Fig. 1B), suggesting that both sequence optimization and poly(A) tailing are necessary for cellular G protein expression. As lipid shells are necessary for mRNA vaccine delivery [43], the RABV-G C sequence was then encapsulated into lipid nanoparticles (LNPs) to become our mRNA vaccine candidate LVRNA001. Briefly, the LVRNA001 construct includes a 5′ cap structure, a 5’ UTR, an open reading frame (ORF), a 3′ UTR and a poly(A) tail. The ORF in this study encodes the glycoprotein (RABV-G) of the CTN-1 strain (GenBank: ACR39382.1). Spherical nanoparticles with a uniform size distribution were observed under an TEM (Fig. 1C).

### Immunogenicity assessment of LVRNA001 in mice

**Fig. 2:**
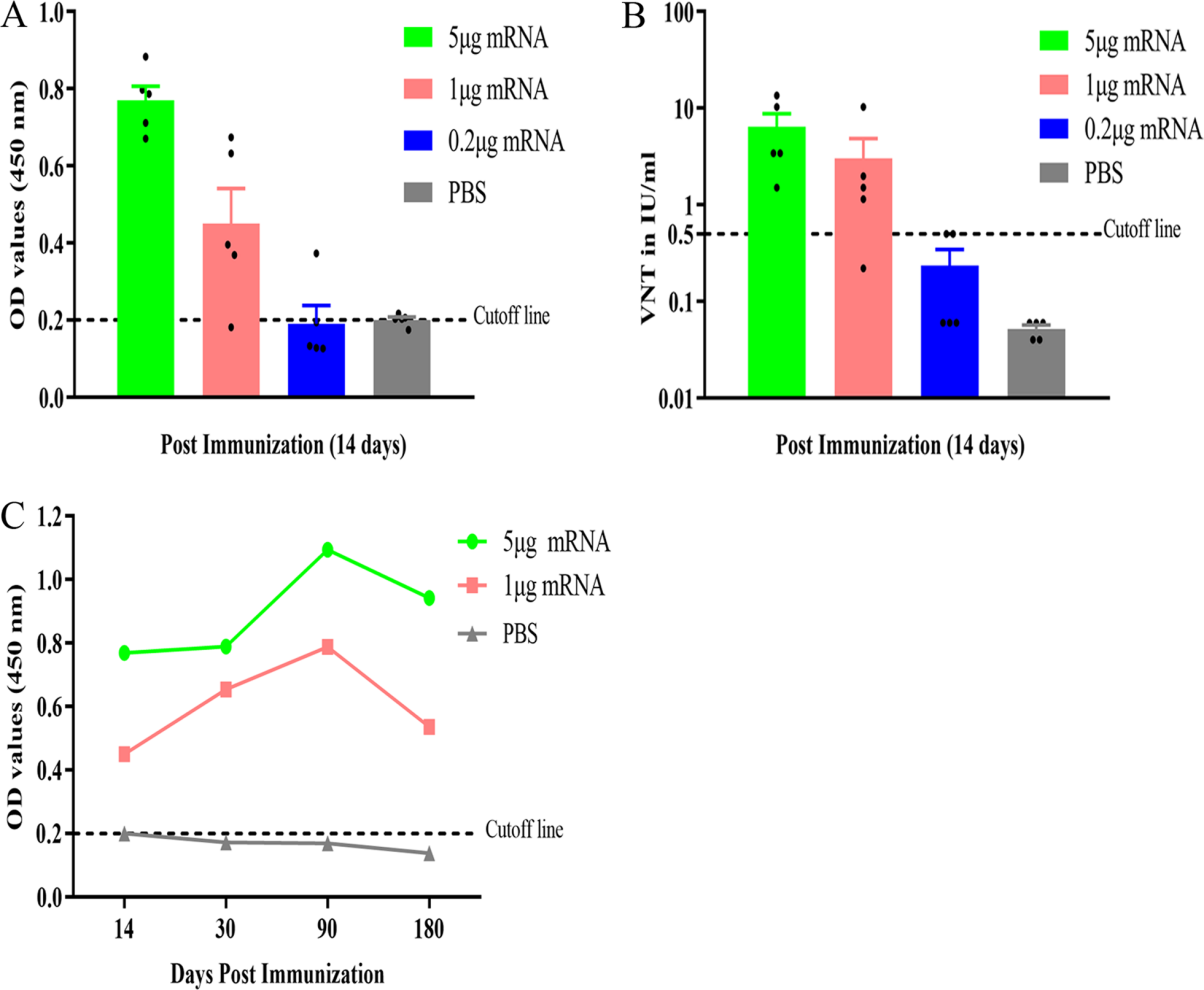
Assays to assess the immunogenicity of LVRNA001 in mice. **A** Mice were immunized via i.m. injection with a single dose of LVRNA001 (0.2 μg, 1 μg or 5 μg) or PBS. At 14 days post-immunization (dpi), serum samples were collected and IgG levels were detected using ELISAs. **B** Neutralizing antibody levels in serum from mice immunized with LVRNA001 (0.2 μg, 1 μg or 5 μg) or PBS were measured by FAVN test. **C** IgG antibody titers in mouse serum were monitored at different time points (0.5, 1, 3, and 6 months) after vaccination with 5 μg or 1 μg of LVRNA001. n = 5/group

To evaluate the immunogenicity of LVRNA001, BALB/c mice were vaccinated via intramuscular (i.m.) injection with LVRNA001 at different dosages: 5 μg, 1 μg, and 0.2 μg. ELISA detected anti-RABV-G IgG levels and demonstrated that the IgG levels induced by LVRNA001 were dose-dependent (Fig. 2A). Specifically, the FAVN test revealed that mice immunized with 5 μg or 1 μg of LVRNA001 achieved neutralizing antibody levels above the threshold concentration of antibody (0.5 IU/ml), considered as protective in humans, dogs and cats [44], whereas mice immunized with 0.2 μg of LVRNA001 or PBS did not achieve this threshold antibody concentration (Fig. 2B). Then, we monitored the IgG antibody titers in the serum of mice at different time points (0.5 months, 1 month, 3 months and 6 months) after vaccination with 5 μg or 1 μg of LVRNA001. We found that both the 5 μg and 1 μg doses of LVRNA001 sustained high levels (> 0.2 ELISA cutoff) of IgG antibody titers at all time points, with levels peaking at 3 months post-immunization (Fig. 2C). Our results indicate that LVRNA001 administered at a dose of 5 μg or 1 μg can effectively induce antibody production in mice and sustain high antibody levels for at least 6 months post-immunization.

### LVRNA001 induced a cellular immune response in mice

**Fig. 3:**
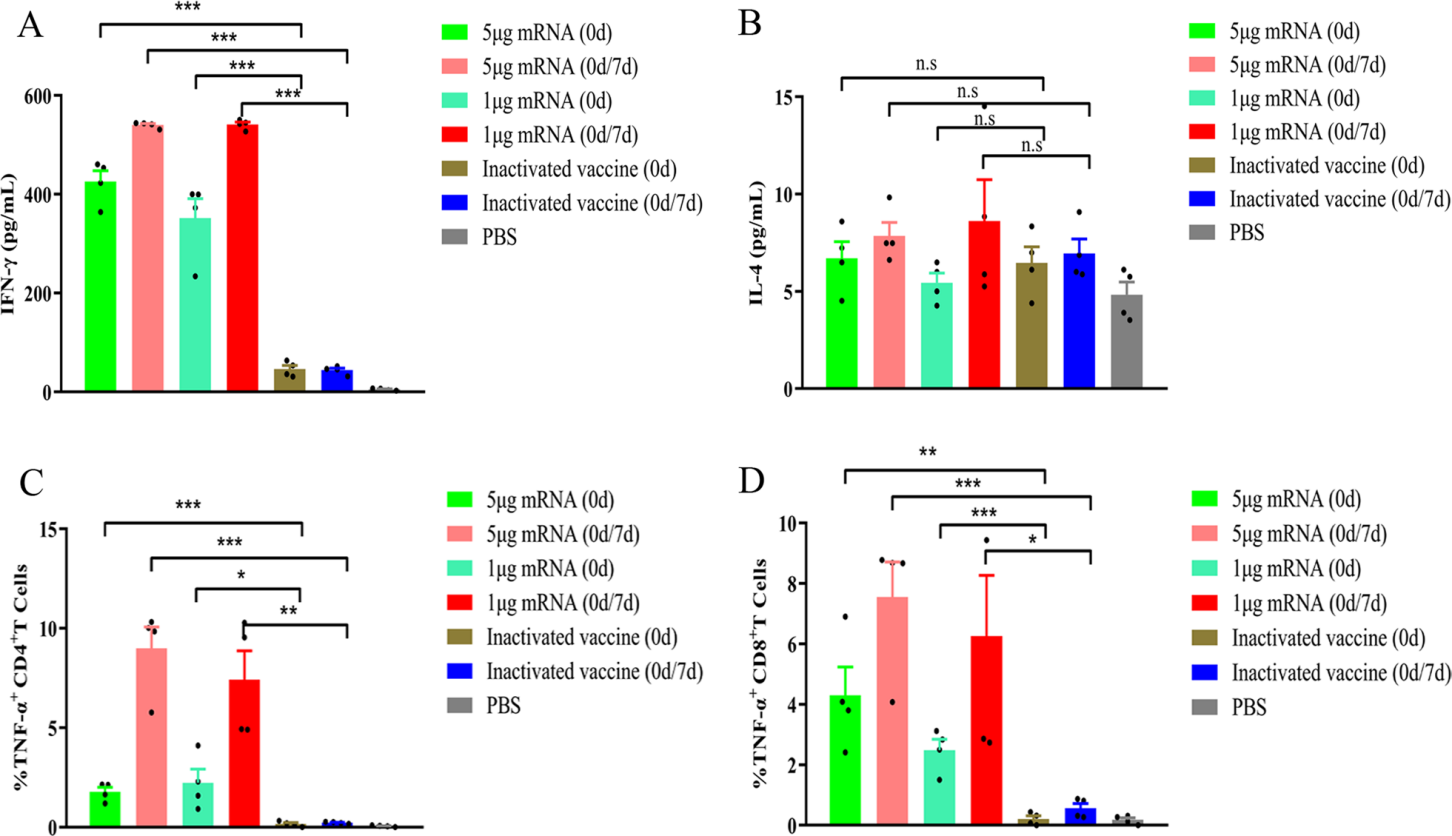
LVRNA001 induced a cellular immune response in mice. Mice received one (0 d) or two inoculations (0d/7d) with LVRNA001 (1 μg or 5 μg), or Rabvac®. PBS served as negative control. **A**, **B** Lymphocytes from the mice in each immunization group were collected. IFN-γ and IL-4 levels in the lymphocytes were detected using ELISAs. **C**, **D** TNF-α-producing CD4^+^ and CD8^+^ T cells from splenic lymphocytes were measured using flow cytometry. n = 4/group. **, p < 0.01. ***, p < 0.001

In mice vaccinated with 5 μg or 1 μg of LVRNA001, the Th1 and Th2 cellular immune responses were detected and characterized (Fig. 3) in comparison with an inactivated vaccine Rabvac®. One (0 d) or two inoculations (0d/7d) with LVRNA001 at either dosage produced significantly higher IFN-γ levels than with PBS or Rabvac® (Fig. 3A). No differences in IL-4 levels were observed between the LVRNA001-immunized groups and the PBS or inactivated vaccine-immunized groups (Fig. 3B). The expression of TNF-α-producing CD4^+^ and CD8^+^ T cells in mice receiving LVRNA001 inoculations was elevated compared to that in mice receiving inactivated vaccine (Fig. 3C–D). These findings imply that LVRNA001 effectively promotes the Th1 cellular immune response in mice.

### LVRNA001 induced a humoral immune response and protected mice against RABV challenge

**Fig. 4:**
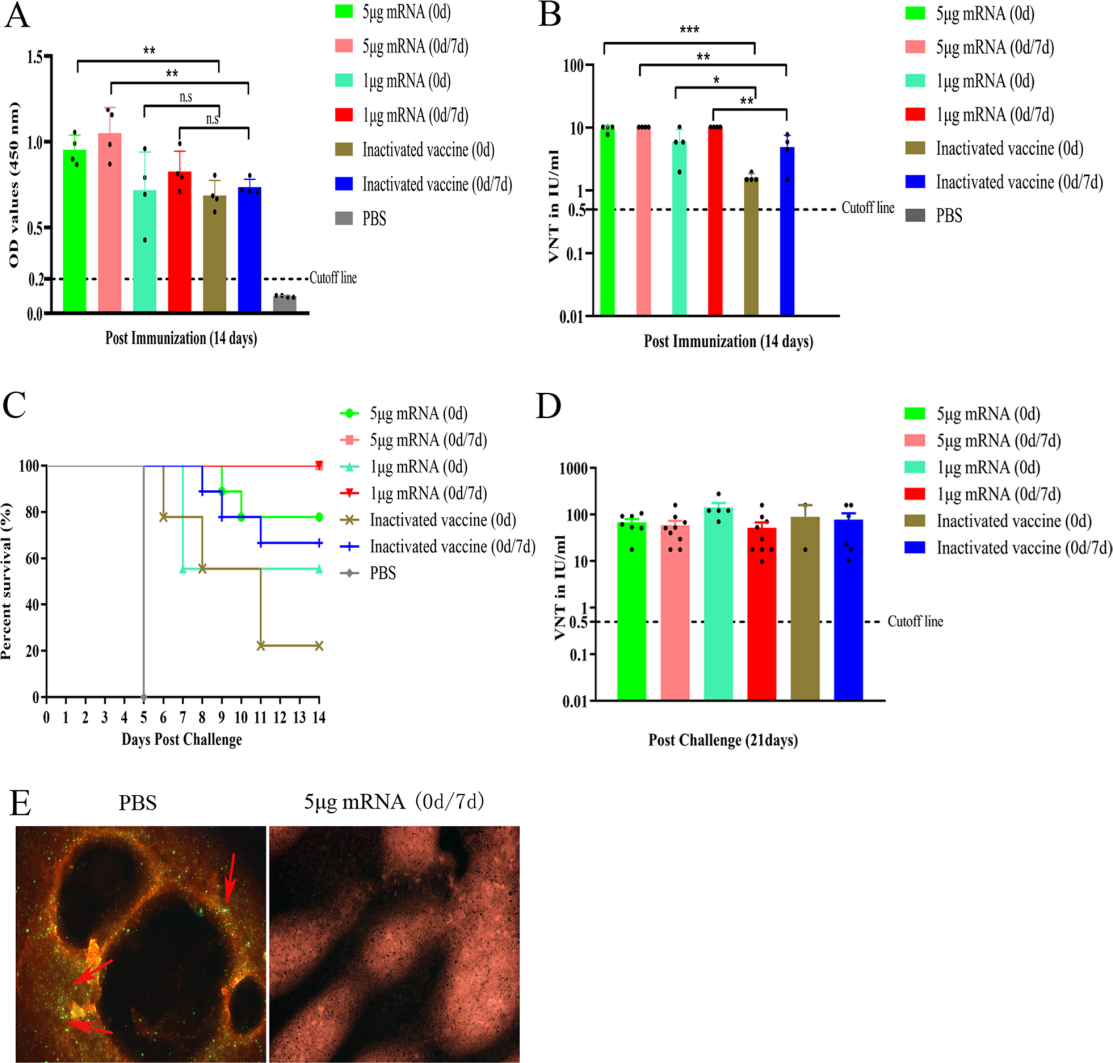
LVRNA001 induced a humoral immune response and protected mice against RABV challenge. Mice were intramuscularly injected with LVRNA001 (1 μg or 5 μg) or Rabvac® (0.1 dose) for once (0d) or twice (0d/7d). PBS served as negative control. **A**–**B** Mouse serum samples were collected on day 14 after first injection, and IgG and neutralizing antibody levels were measured via ELISAs and FAVN tests, respectively. n = 4/group. **C** At 14 dpi, mice were intracerebrally challenged with 50-fold LD_50_ of the virulent RABV-CVS-24 strain. The survival rates of the infected mice were monitored until 14 days post-challenge. n = 9/group. **D** Serum samples of surviving mice from C were collected at 21 days post-challenge, and the FAVN test was used to detect neutralizing antibody levels. **E** Direct immunofluorescence detection of RABV in dead mouse brain by using a RABV-specific antibody at day 21 post-challenge

ELISAs revealed that at 14 days post-immunization (dpi), the IgG levels in the mice that received LVRNA001 were above the cutoff line and slightly higher than those in mice immunized with inactivated vaccine (Fig. 4A). The overall neutralizing antibody levels at 14 dpi reflected the same pattern (Fig. 4B). Mice were then intracerebrally challenged with RABV, and the survival rates (14 days post-challenge) were evaluated (Fig. 4C). We found that administering two doses (0d/7d) of LVRNA001 at either 5 μg or 1 μg resulted in 100% of the mice surviving the challenge, whereas a single injection (0d) of LVRNA001 at either 5 μg or 1 μg resulted in only 77.8% and 55.6% survival, respectively. In addition, the survival rates were also evaluated in the mice that received inactivated vaccine. The results showed that 22.2% of these mice injected once (0d) survived, while 66.7% those injected twice (0d/7d) survived. All mice in the PBS group died within 5 days. At 21 days post-challenge, the neutralizing antibody levels in the surviving mice were measured. Both LVRNA001 and the inactivated vaccine induced high antibody levels (≥ 0.5 IU/ml), indicating potential humoral responses and protective efficacy (Fig. 4D). Additionally, indirect immunofluorescence did not show the presence of RABV in the brain tissue of mice receiving two inoculations (0d/7d) of 5 μg of LVRNA001, while the virus was detected in PBS-treated mouse brain tissue (Fig. 4E). These data indicate that LVRNA001-induced antibodies can efficiently prevent viral replication in mice.

### Extension of the dosing interval promoted LVRNA001 effectiveness in mice

**Fig. 5:**
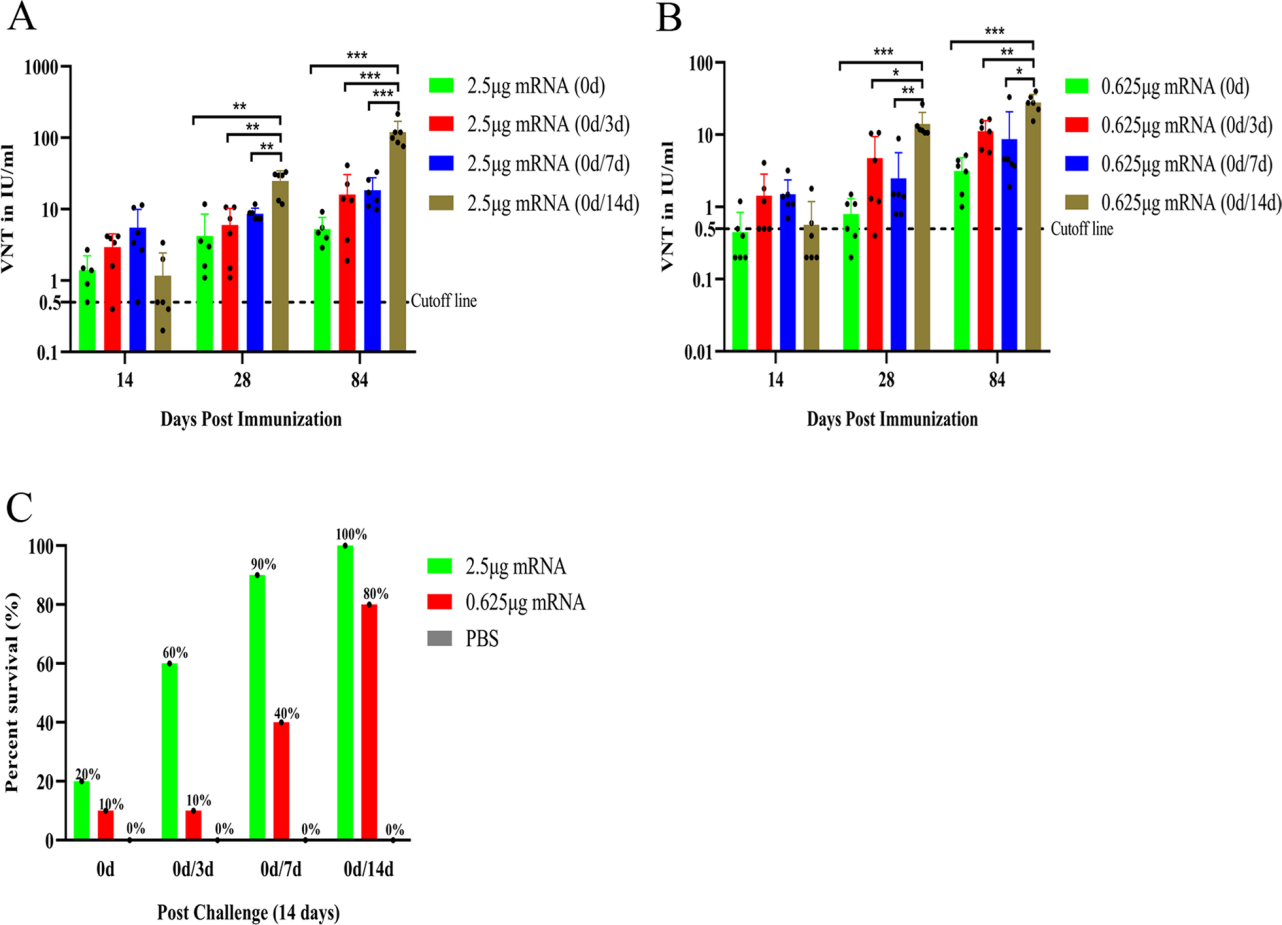
Extension of the dosing interval promoted LVRNA001 effectiveness in mice. Mice were immunized with different doses of LVRNA001 (2.5 μg or 0.625 μg) or PBS. A second dose of LVRNA001 was administered at 3 days (0d/3d), 7 days (0d/7d), and 14 days (0d/14d) after first immunization. (**A**–**B**) Mouse serum samples were collected at 14, 28 and 84 dpi, and neutralizing antibody levels were measured by FAVN tests. n = 6/group. *, p < 0.05. **, p < 0.01. ***, p < 0.001. **C** Mice were intracerebrally challenged with 50-fold LD_50_ of the virulent RABV-CVS-24 strain, the survival rates at 14 days post-challenge were calculated. n = 10/group

Determining the dosing interval for a two-dose vaccine regimen is critical. We therefore compared the serological responses and survival rates of the mice inoculated with a second dose of LVRNA001 at 3 days, 7 days, and 14 days after their first immunization (Fig. 5). Regardless of whether the dosages were larger (2.5 μg, Fig. 5A) or smaller (0.625 μg, Fig. 5B), the neutralizing antibody levels at 28 dpi and 84 dpi were significantly increased in the 14-day interval group versus the 3-day interval and 7-day interval groups. However, no significant difference between any of the groups was observed at 14 dpi. The survival rates of the mice at 14 days post-challenge were also observed to be higher in mice immunized a second time after 14 days than that of the mice immunized again after 3 or 7 days (Fig. 5C).

### LVRNA001 provided effective protection to dogs infected with RABV

**Fig. 6:**
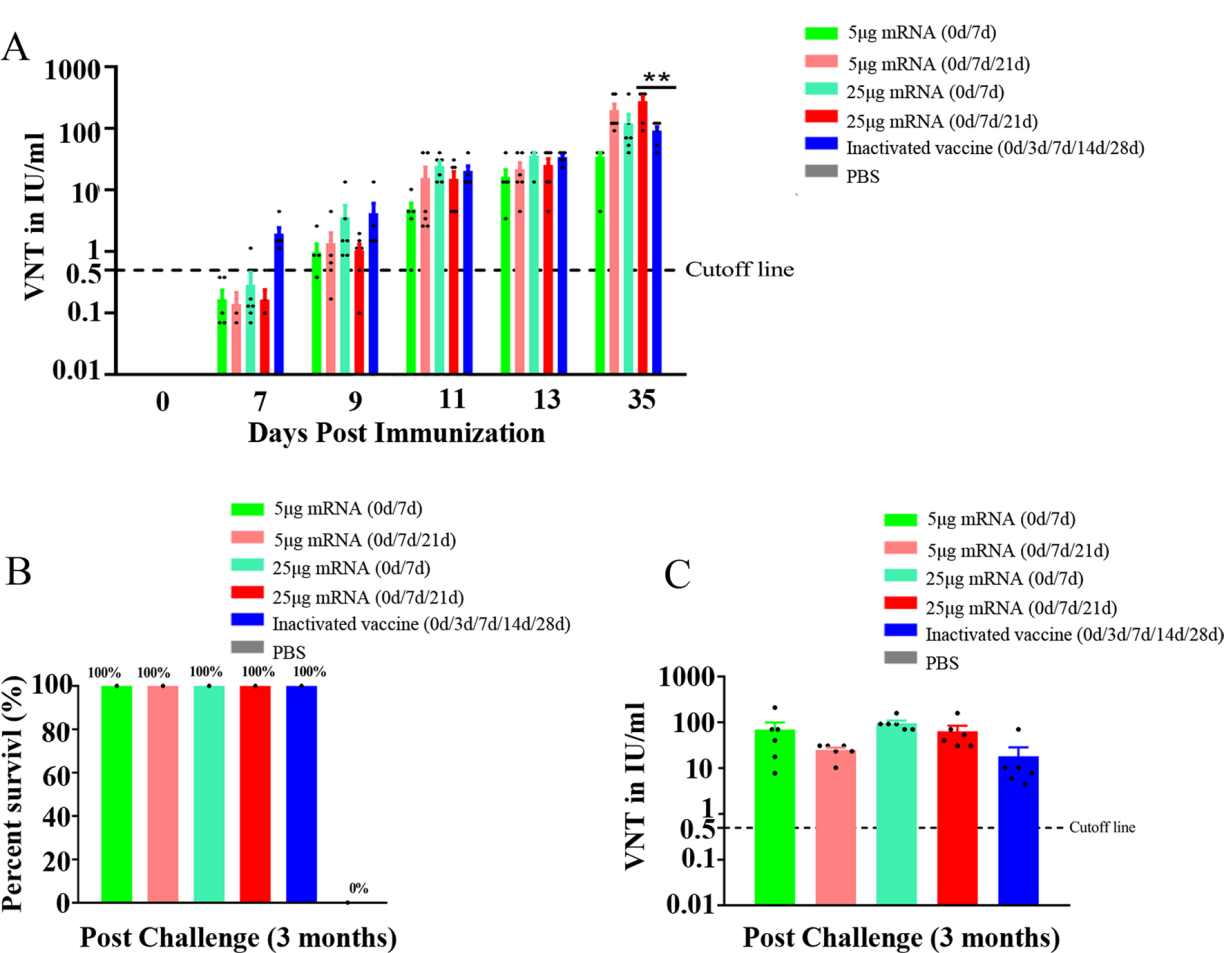
Pre-exposure immunization of LVRNA001 efficiently protected dogs from live virus infection. Dogs were intramuscularly immunized twice (0d/7d) or three times (0d/7d/21d) with LVRNA001 (5 μg or 25 μg dosages), five times (0d/3d/7d/14d/28d) with inactivated vaccine, or PBS. **A** Serum samples from dogs were collected at 7, 9, 11, 13, and 35 dpi, FAVN tests were conducted to detect neutralizing antibody levels. **B** Dogs were challenged with 50-fold LD_50_ of the virulent RABV-BD06 strain by i.m. injection at 35 dpi. The survival rates were calculated at 3 months post-challenge. **C** Neutralizing antibody levels in the surviving dogs at 3 months post-challenge were measured using FAVN test. n = 6/group

Dogs were intramuscularly immunized with LVRNA001 (5 μg or 25 μg dosages) for either two doses (0d/7d) or three doses (0d/7d/21d). Antibody response following pre-exposure immunization against RABV was measured. For all dosage and interval combinations, the induced neutralizing antibody levels exceeded 0.5 IU/ml at 9, 11, 13, and 35 dpi (Fig. 6A). Dogs vaccinated with 25 μg of LVRNA001 (0d/7d/21d) induced the production of more neutralizing antibodies than dogs immunized with inactivated vaccine (0d/3d/7d/14d/28d) at 35 dpi; no neutralization activity was detected in the PBS group throughout the experiment (Fig. 6A). These dogs were then infected with 50-fold LD_50_ of the virus by i.m. injection at 35 dpi, and the survival rates (3 months post-challenge) were compared. Dogs that received 2 doses or 3 doses of LVRNA001 (5 μg or 25 μg, 0d/7d or 0d/7d/21d) demonstrated 100% survival (Fig. 6B), as did the dogs that received 5 doses of inactivated vaccine (0d/3d/7d/14d/28d). In contrast, all dogs in the PBS group died by end of the 3-month observation period (Fig. 6B). Neutralizing antibody levels were measured in the surviving dogs at 3 months post-challenge, the results showed that all four LVRNA001-immunized groups and the inactivated vaccine group sustained high levels of antibody production (Fig. 6C).

**Fig. 7:**
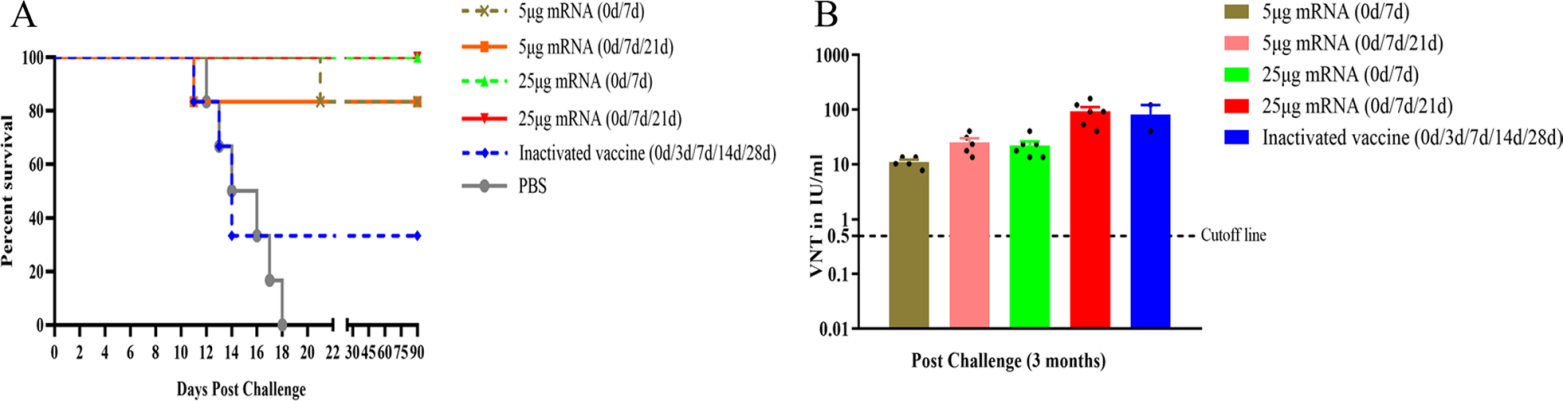
Post-exposure injection of LVRNA001 to dogs exerted protective efficacy. Dogs were challenged with 50-fold LD_50_ of the virulent RABV-BD06 strain, six hours later, they were immunized with two doses (0d/7d) or three doses (0d/7d/21d) of LVRNA001 (5 μg or 25 μg), five doses (0d/3d/7d/14d/28d) of inactivated vaccine, or PBS, followed by clinical observation. **A** The numbers of surviving dogs were recorded and survival rates were calculated. **B** Neutralizing antibody levels in surviving dogs were analyzed via the FAVN test at 3 months post-challenge. n = 6/group

To further assess effect of LVRNA001 on protective efficiency in dogs, post-exposure immunization against RABV was conducted. Dogs were first infected (i.m.) with 50-fold LD_50_ of the virus, six hours later, inoculated twice (0d/7d) or three times (0d/7d/21d) with LVRNA001 (25 μg or 5 μg dosages). Three months post-infection, the survival rates of the dogs immunized with 25 µg of LVRNA001 (0d/7d or 0d/7d/21d) were 100%, and the survival rates of the 5 μg dosage groups were 83.33%. In contrast, only 33.33% of dogs vaccinated with inactivated vaccine (0d/3d/7d/14d/28d) survived, and all dogs given PBS died (Fig. 7A). Analysis of the neutralizing antibody levels 3 months post-challenge found that all immunized dogs sustained high neutralizing antibody levels (> 0.5 IU/ml) (Fig. 7B).

## Discussion

In the current study, an optimized mRNA construct (LVRNA001) expressing the RABV-G protein was designed and developed in vitro. In vivo, LVRNA001 effectively induced antibody production and stimulated cellular immune responses. Inoculation with LVRNA001 provided mice with protective immunity against RABV infection, the highest protection observed when mice received two doses of LVRNA001 over an extended dosing interval of 14 days. Protection against infectious challenge was also stringently verified in dogs, as dogs administered with LVRNA001 produced protective antibody levels and demonstrated 100% survival at 3 months post-infection.

RABV-G is an important viral component that induces the host antibody response, allowing it to serve as a main immunogen in rabies vaccines [45, 46]. In this study, we optimized the RABV-G nucleotide sequence in order to achieve high level expression of the protein. In addition to an optimized open reading frame (ORF), ideal RABV-G mRNA construct requires careful consideration of other structural component. The 5′ and 3′ UTR elements flanking the coding sequence are vital to mRNA stability and translation, also can elevate the half-life and expression of mRNA in vaccines [47, 48]; the poly(A) tail is involved in the regulation of mRNA translation and stability [49]; and the GC content of the construct, when enriched, has been found to increase steady-state mRNA levels and protein expression [50, 51]. For LVRNA001, the GC content of the G protein expression nucleotide sequences was raised to 56% in comparison with the original GC content of 46%. Moreover, a poly(A) tail with 100A was introduced. The resulted construct manifested a high level of RABV-G expression in both cellular and animal studies.

In the immune response, macrophages and dendritic cells activate a Th1 response by secreting cytokines such as IFN-γ and TNF-α, and B cells favor a Th2 response by releasing other cytokines, including IL-4 [52–54]. Th1-type cytokines are hallmarks of both innate and adaptive cell-mediated immunity, whereas allergies are regarded as a Th2-weighted imbalance because allergen-specific T cells invariably have a Th2 phenotype [55]. In our study, LVRNA001 stimulated significantly more Th1 cytokines (IFN-γ and TNF-α) than the inactivated vaccine in mice, but the IL-4 levels were not different between LVRNA001 and inactivated vaccine treatment, suggesting that LVRNA001 promotes a strong Th1 cellular immune response, which is advantageous for vaccination. Additionally, numerous studies have evidenced that Th1 cells are critical in the clearance of RABV from central nervous system [56, 57]. Therefore, on one hand, the resulting immune reactions can produce memory immune cells and neutralizing antibodies; on the other hand, these reactions do not cause adverse inflammatory effects. On a separate note, extending the dosing interval contributed to LVRNA001 effectiveness against RABV infection in mice. This is in line with Payne et al.’s research, which found that extended dosing intervals boosted serologic responses to SARS-CoV-2 [58]. All of these findings suggest that LVRNA001 may serve as a promising and efficient RABV-G mRNA vaccine to allow for significant improvement in vaccine supply.

With dogs, pre-exposure immunization of LVRNA001 induced neutralizing antibody levels as high as or even more than did by classic inactivated vaccine (Fig. 6). In the post-exposure experiments, LVRNA001 demonstrated a > 80% survival rate; however, this survival rate was not observed in dogs immunized with the inactivated vaccine (Fig. 7). It was noticed that animal death in the inactivated vaccine group happened within 14 days after viral challenge, when the 5-dose immunization program was only partially run (Fig. 7A). We also speculate that virus variation played a role in this particular experiment: dogs were infected by the RABV-BD06 strain, while the inactivated vaccine was made from RABV-aGV strain. RABV-G protein sequence homology between BD-06 and aGV strains is only 88%, meanwhile homology between BD-06 and CTN-1 (mRNA vaccine sequence) is 94%. In comparison to its human post-exposure application, inactivated vaccine inoculation is usually accompanied by usage of immunoglobulin against Rabies virus in order to achieve maximal anti-rabies efficacy.

Most rabies vaccines in current medical usage are inactivated viruses derived from cell culture with complicated manufacturing processes and quality control procedures, leading to an unstable market supply and product safety concerns. Moreover, individuals need 4–5 doses of inactivated vaccine to achieve efficient protection. In this report, we demonstrated that immunization with two doses of an mRNA vaccine can achieve equal or better efficacy in mice and dogs without observing any adverse clinical effects. From a manufacturing perspective, large-scale production of mRNA vaccines is less time-consuming and more effective than inactivated vaccines. Taking together, we believe that LVRNA001 could be a good vaccine candidate for future rabies control. However, as our current study focused on only mice and dogs, clinical studies are needed to further evaluate the safety and efficacy of this mRNA vaccine in human.

## Data Availability

The data and materials used in the current study are available from the corresponding author on reasonable request.

## Abbreviations

RABV: Rabies virus
mRNA: Messenger RNA
RABV-G: Rabies virus glycoprotein
LD_50_: 50-Fold lethal dose 50
i.m.: Intramuscular
PBS: Phosphate buffered solution
DMEM: Dulbecco’s modified Eagle’s medium
DSPC: 1,2-Distearoyl-sn-glycero-3-phosphocholine
TEM: Transmission electron microscopy
FBS: Fetal bovine serum
IFN-γ: Interferon-γ
IL-4: Interleukin-4
VNA: Viral neutralizing antibody
FAVN: Fluorescent antibody virus neutralization
TCID_50_: 50% Tissue culture infectious dose
BSA: Bovine serum albumin
SD: Standard deviation
LNPs: Lipid nanoparticles
ELISA: Enzyme-linked immunosorbent assays
dpi: Days post-immunization
